# Evaluation of Gene Expression Classification Studies: Factors Associated with Classification Performance

**DOI:** 10.1371/journal.pone.0096063

**Published:** 2014-04-25

**Authors:** Putri W. Novianti, Kit C. B. Roes, Marinus J. C. Eijkemans

**Affiliations:** Biostatistics & Research Support, Julius Center for Health Sciences and Primary Care, University Medical Center Utrecht, Utrecht, The Netherlands; Deutsches Krebsforschungszentrum, Germany

## Abstract

Classification methods used in microarray studies for gene expression are diverse in the way they deal with the underlying complexity of the data, as well as in the technique used to build the classification model. The MAQC II study on cancer classification problems has found that performance was affected by factors such as the classification algorithm, cross validation method, number of genes, and gene selection method. In this paper, we study the hypothesis that the disease under study significantly determines which method is optimal, and that additionally sample size, class imbalance, type of medical question (diagnostic, prognostic or treatment response), and microarray platform are potentially influential. A systematic literature review was used to extract the information from 48 published articles on non-cancer microarray classification studies. The impact of the various factors on the reported classification accuracy was analyzed through random-intercept logistic regression. The type of medical question and method of cross validation dominated the explained variation in accuracy among studies, followed by disease category and microarray platform. In total, 42% of the between study variation was explained by all the study specific and problem specific factors that we studied together.

## Introduction

Microarray gene expression technology continues to be used to obtain more understanding of the mechanisms of human diseases. The statistical analysis of microarray data may be challenging, with the inherent risk of finding a false positive result due to the high dimensional nature of the data. Common flaws in the three distinctive goals for the statistical analysis of microarray data (e.g. differential expression, class discovery (unsupervised), and class prediction (supervised)) have been found [1]. Inconsistency in the results of microarray analyses within the same dataset unfortunately has also been reported, especially for class prediction [2]. The variability of the reported classification accuracies may be due to the variation in the methods used to build the classification model, e.g. the type of classification model, cross validation and gene selection strategy [3]. Additionally, the performance of a predictive model may also depends on characteristic of the microarray data [4].

Most of the studies evaluating classification performance have concentrated on classification of cancer patients. In general, non-cancer diseases have received low attention in the gene expression literature, maybe because they have more varying levels of complexity than cancers. However, to test the hypothesis that disease complexity influences classification performance, it may be beneficial to use a variety of studies on non-cancerous diseases, instead of cancer studies.

This study focuses on the factors that might be associated with the accuracy of classification models on gene expression datasets, namely the type of disease, the medical question, sample size, the number of genes, the gene selection method, the classification method and cross validation techniques, using published studies outside of the field of cancer. Although it was evaluated differentially, we noticed that there is an overlap of the aforementioned study factors with the observed factors by the MAQC II study [3] that is focused in the field of cancer, i.e. the number of genes, the gene selection method, and the classification method. In the case of non-cancerous diseases, those study factors may also affect the performance of classification method. The results of this study may contribute to understand the dependency of the performance of a classification model on the characteristics of gene expression data as well as the techniques used to build the model.

## Materials and Methods

### Literature search and data extraction

We searched microarray gene expression studies through PubMed (US National Library of Medicine National Institute of Health) for relevant papers. Applied studies in which the investigator aimed to build supervised models based on microarray gene expression experimental data were primarily of interest. The studies that 1) were published in methodological journals 2) focused on cancer 3) were published before 2005 4) had non-human species as experimental objects 5) were not written in English or 6) were categorized as review papers, were not included. For the details of the search strategy and keywords see Material S1.

The search strategy and selection of studies satisfied the general methods for Cochrane reviews. The following details were recorded from each selected study: classification performance, disease type, medical question (diagnosis, prognosis or response-to-treatment), microarray platform (one- or two-color system), total sample size, sample size per group, cross validation technique (single or nested loop cross validation), gene selection technique (filter, wrapper, or embedded), classification method(s), and the number of genes. The selected studies had evaluated the classification models in diverse ways, e.g. accuracy or misclassification error, sensitivity and specificity, positive and negative predictive values, as well as AUC. The accuracy was then used to represent the classification performance, since it is the most commonly used measure by the selected studies and feasible to be produced in some studies when the information about the accuracy lacked.

Sample size was recorded as the sample size in the training set, used to build the model. The degree of class imbalance was measured by dividing the number of samples in the majority class with the total sample size in the training set. Due to the diversity of cross validation methods used, we grouped the cross validation techniques into single and nested cross validation. The studies that used cross validation for both model assessment and model selection were grouped into nested cross validation. Otherwise, it was regarded as single cross validation.

Some classification methods have the ability to automatically handle the curse of dimensionality (p>>n), but others need a gene selection step to reach a lower dimension before applying the classification method. Some of the studies selected genes univariately based on a statistic passing a threshold for selection or the top-K genes to feed the classifier. In other studies, the gene selection method was aimed at finding an optimal set of genes by stepwise iterating between selection and classifier building. Thus, we grouped the gene selection technique based on their interaction with the classifier, namely filter (e.g. univariate selection), wrapper (e.g. stepwise optimization of the selected gene set), and embedded (e.g. penalized likelihood regression). Grouping was also done on the classification method into two categories, depending on their ability to detect interactions between genes. Genes can be activated independently but also be activated through the activation of other genes. Due to this phenomenon, the classification methods that can automatically model interactions are expected to have better performance than those who cannot, at least in some studies. The methods that could detect the interaction (referred to as “interaction classifiers”) in our review were tree-based methods, logistic regression, support vector machines (SVM), *k*-Nearest Neighbours (*k*NN), artificial neural networks (ANN), and weighting voting methods. Meanwhile, discriminant analysis, prediction analysis of microarray (PAM), compound covariate predictor, nearest centroid, and LASSO were classified into the group of methods that could not automatically detect interactions (called “non-interaction classifiers”).

Among the selected studies, we found 34 different disease types. The diseases were categorized according to SNOMED (http://eagl.unige.ch/SNOCat/) producing 16 categories. Further re-categorization was done to establish etiology-based disease groups. As a result, we obtained 6 disease types: inflammatory disorder, immune disease, degenerative disease, infection, mental disorder and other (i.e. obesity and acute lung injury). See Material S2 for grouping details.

### Data analysis

The forty eight selected studies yielded sixty one classification gene expression models. The number of observed classification models is higher than the number of selected studies since some studies had built more than one classification model. We considered the data to be clustered data, where the selected studies act as clusters. Further, in each study, we treated the accuracy as a grouped binomial variable, for which we have the number of samples that are correctly and incorrectly classified. The data structure is visualized in Table 1. The logistic random effect meta-analysis is a natural choice to handle this type of data [5]. The logistic random effects model is the generalization of the linear mixed effects model to binomial outcome data using a sigmoid link function

**Table 1:** Overview of the studied data.

Study	Classification model(s)	Study factor 1	…	Study factor 8	Classfication model accuracy
1	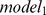				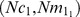
2	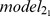				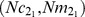
3	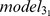				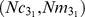
4	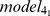				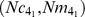
5	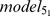				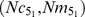
6	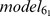				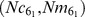
7	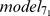				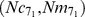
7	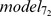				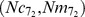
7	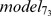				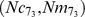
7	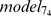				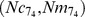
…	…	…	…	…	…
45	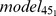				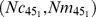
46	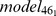				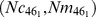
47	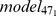				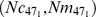
48	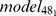				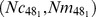

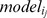
: Classification model j in study i.

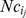
: The number of correct classified sample(s) based on the classification model j in study i.

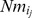
: The number of miss-classified sample(s) based on the classification model j in study i.

As the accuracy is well known to be biased towards the majority class, the random intercept logistic model was corrected by the class imbalance level, which was always included in the meta-regression model. For the 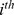 study factor, the random effects model is written as
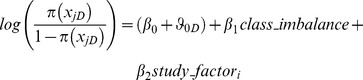
where 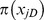 is the probability of a sample *j* in dataset *D* to be correctly classified and 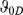 is the random intercept with respect to dataset *D*, in which 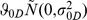. Multivariable evaluation of study factors was also done by a backward elimination approach. In each backward step, two nested models, with and without a particular study factor, were compared by Akaike's information criterion (AIC).

The explained-variation of the model accuracy was then calculated on the log-odds scale using the random effects variance of the study factor. The variation explained by all study factors together was calculated based on the relative difference between the random intercept variances of the null model 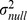 and the full model 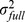, divided by 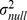. The full model is the logistic random intercept model with all study factors as covariates. We also evaluated the explained variation of each factor relative to the full model. The relative contribution in explained variation by the 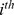 factor to the full model was calculated by
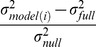
(1)where 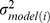 is the variance of the model based on all factors except the 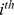 factor 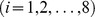 and 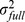 is the variance of the model based on all factors. All analyses have been done in R software (Material S3). The glmer function from lme4 package was used to analyse the data [6].

## Results

### Summary of study characteristics

**Figure 1:**
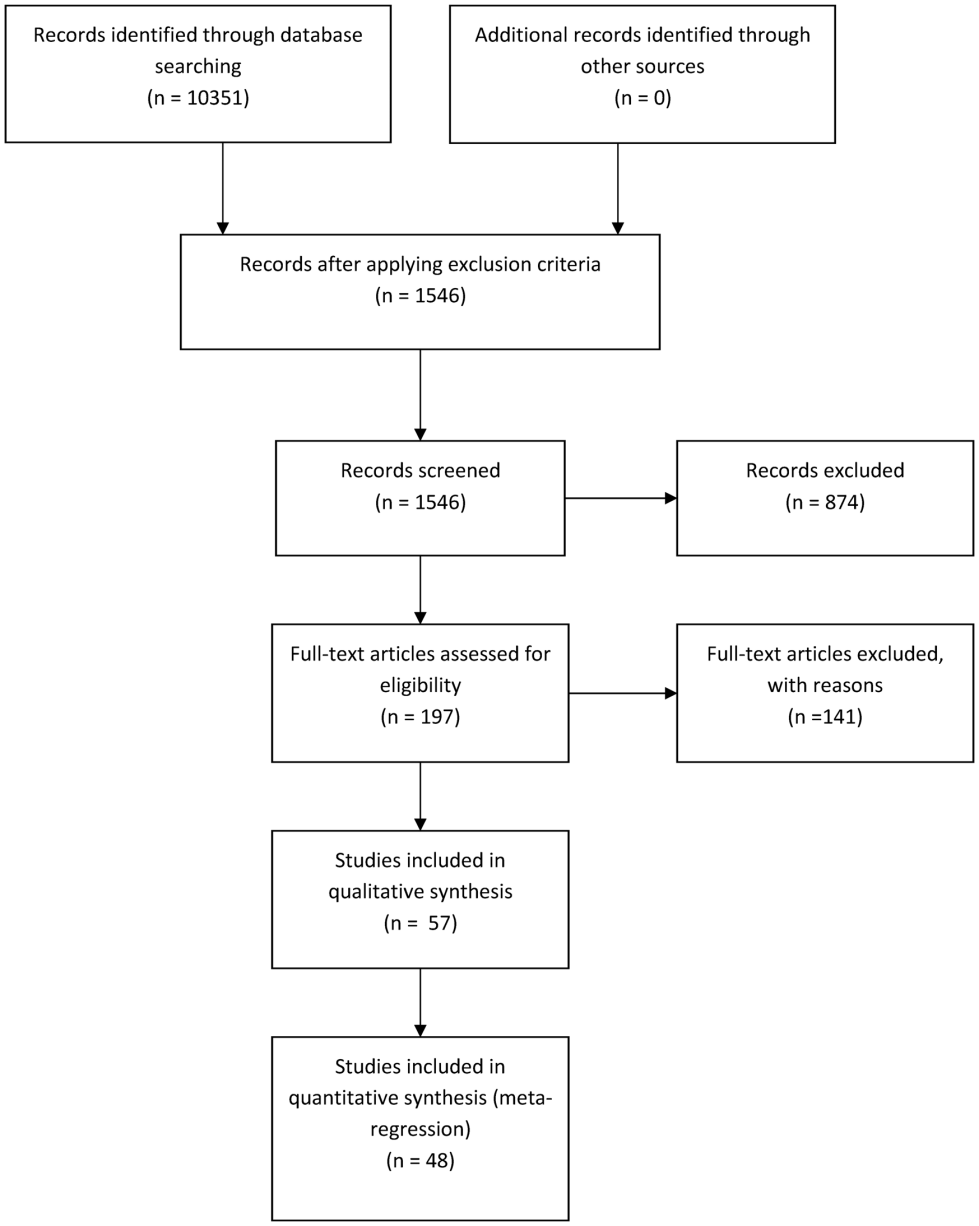
The PRISMA workflow diagram of the literature review search. The diagram represents the process of literature review search. The details for each step can be found in the Material S1.

The automated search strategy yielded over a thousand papers. The first screening was done by examining the title and abstract, and yielded 197 papers to be fully reviewed, which resulted in 57 papers that met all the criteria (last search on September 20, 2013). The PRISMA workflow diagram of the systematic literature review [7] is provided in Figure 1.

For further statistical analyses, we selected the 48 studies [8]–[55] that mentioned accuracy as their classification performance measurement. Because some studies had used more than one classification method, the evaluation of factors influencing accuracy was based on 61 classification models. The basic characteristics of the selected studies are described in Table 2.

**Table 2:** Characteristics of 48 fully reviewed studies.

Study characteristics	Number of studies
**Year**	
2005–2007	15
2008–2010	19
2011–2013	14
**Disease Type**	
Inflammatory disorder	17
Immune disease	5
Degenerative disease	6
Infection	12
Mental disorder	5
Other	3
**Microarray color system**	
One-color	35
Two-color	13
**Medical question**	
Diagnostic	29
Prognostic	6
Response-to-treatment	13
**Cross validation**	
Single	19
Nested	29
**Gene selection**	
Filter	13
Wrapper	16
Embedded	19
**Classification method** *	
Interaction	37
No-interaction	24

*Some studies used more than one classifier.

Within the search period, the number of classification studies that had used microarray technology outside the field of cancer tended to increase with calendar time, and the one color system array (35/48) was mostly used, compare to the two-color system (13/48). We found 34 different diseases, predominantly the inflammatory disorder and infection disease groups, i.e. 17 (35%) and 12 (32%) studies, respectively. The diagnostic problem is the most common medical question addressed by microarray gene expression supervised learning. Classification models were built by either the single (19/48) or the nested (29/48) cross validation technique. The search result shows that there is no clear preference in dimensionality reduction technique among the selected studies. With regards to the classification methods, we notice that SVM (24%) and PAM (21%) are the most commonly used methods. However, when we grouped the classification methods based on their ability to detect interaction between genes, there appeared to be no clear preference for no-interaction or interaction classifiers.

### Meta-regression

Table 3 shows the result of individual evaluation for each factor by random effects logistic regression. A model with “class imbalance level” as a fixed effect is considered as the null model. No model with an additional fixed effect is better than the null model. The multivariable model by backward evaluation is summarized in Table 4. We started with the full model which consists of nine study factors. The backward elimination resulted in four study factors that are associated with the performance of a classification method, namely the color system, medical question, cross validation technique, and gene selection method (Table 4). We refer this model as a final meta-regression model. Although the final model would improve without the “class imbalance level” (shown by the lower AIC value if the classification model is excluded from the random effect model), we keep this factor in the logistic model as a correction as stated in the Methods section.

**Table 3:** Individual random intercept logistic regression.

Study factor	Df	AIC	P value
Class imbalance level	1	142.3	0.18
Sample size	1	142.9	0.23
Microarray platform (color system)	1	142.8	0.22
Medical question	2	144.1	0.33
Disease type	5	148.3	0.66
Cross validation technique	1	144.0	0.66
Gene selection method	2	144.7	0.45
Classification method	1	144.3	0.90
The number of genes in final model	1	144.3	0.78

**Table 4:** Backward elimination in multiple random intercept logistic regression.

Step	Study factors on the model	Df	AIC+	P value
1	Class imbalance level	1	155.95	0.128
	Sample size	1	153.65	0.928
	Microarray platform (color system)	1	154.85	0.271
	Medical question	2	160.58	0.011
	**Disease type** *	**5**	**149.10**	**0.629**
	Cross validation technique	1	157.56	0.048
	Gene selection method	2	152.72	0.582
	Classification method	1	153.64	0.950
	The number of genes in final model	1	153.91	0.602
2	Class imbalance level	1	148.52	0.233
	**Sample size** *	**1**	**147.10**	**0.993**
	Microarray platform (color system)	1	150.62	0.061
	Medical question	2	151.90	0.033
	Cross validation technique	1	150.80	0.054
	Gene selection method	2	148.90	0.149
	Classification method	1	147.18	0.773
	The number of genes in final model	1	147.61	0.476
3	Class imbalance level	1	146.53	0.232
	Microarray platform (color system)	1	149.07	0.046
	Medical question	2	149.90	0.033
	Cross validation technique	1	149.41	0.038
	Gene selection method	2	147.63	0.104
	**Classification method** *	1	**145.19**	**0.766**
	The number of genes in final model	1	145.62	0.473
4	Class imbalance level	1	144.58	0.239
	Microarray platform (color system)	1	147.32	0.042
	Medical question	2	147.99	0.033
	Cross validation technique	1	147.85	0.031
	Gene selection method	2	145.77	0.101
	**The number of genes in final model** *	1	**143.76**	**0.451**
5	Class imbalance level	1	142.76	0.315
	Microarray platform (color system)	1	145.84	0.043
	Medical question	2	145.99	0.044
	Cross validation technique	1	146.02	0.039
	Gene selection method	2	144.08	0.115

+ The AIC of multivariable random effect logistic regression model if the corresponding study factor is deleted. The AIC in the full model is 155.7.

*The study factors gave the lowest AIC and was excluded from the model for the next step.

**Figure 2:**
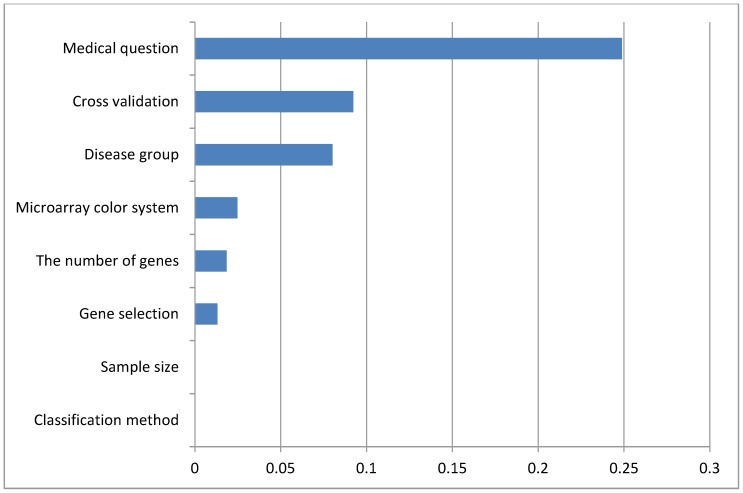
The relative explained-variation of study factors. The x-axis represents the relative explained variation for each study factor, while the y-axis shows the study factors. Table S2 provides more details on the relative explained-variation of each study factor.

The relative contribution of each study factor to the explained-variation of the full model is shown in Figure 2. The medical question has a large relative explained-variation (25%), followed by cross validation technique (9.2%), disease group (8.0%), microarray color system (2.5%), the number of genes in the final classification model (1.8%), and gene selection technique (1.31%). In total, all study factors together explained 41.9% of the between study variation in the null model.

## Discussion

This study was conducted on 48 selected papers that were published between 2005 and 2013 and identified through the PubMed repository. Targeted keywords efficiently selected the relevant papers, among thousands of published microarray classification studies. We aimed to assess the influence of study and method specific determinants of classification model accuracy outside the field of cancer, by analysing eight factors through random effects meta-regression. The accuracy is used as a representation of classification model performance due to the availability of the information in the majority of the selected study. The accuracy is a well-known rough measurement for the performance of a classification model. Especially in highly imbalanced datasets, accuracy may yield overoptimistic results, because a classification model might easily send all samples to the majority class. The class imbalance should therefore be taken into account when interpreting the prediction accuracy [1], and a meaningful classification model necessarily should have higher accuracy than the proportion of the majority class. Unfortunately, the classification models in [9], [30], [35], [44] have lower accuracy than their level of class imbalance (Figure S1). Other measurements, such as Mathew's correlation coefficient (MCC), might be less affected by the class imbalance level. However, it was unfeasible to have all information that is necessary to calculate MCC in all selected studies. To deal with the problem of class imbalance when using accuracy, we corrected our random effects models with the class imbalance level. We then expect that this correction will compensate for the drawback of using accuracy.

**Figure 3:**
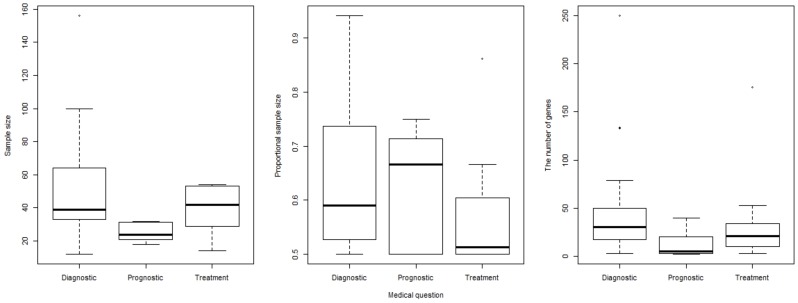
Boxplot of the “Medical question” study factor with respect to sample size on training data, proportional sample size (class imbalance level), and the number of genes included in the final classification models.

The main finding of this study is that four factors were associated with the classification accuracy. The clinical problem (i.e. diagnostic, prognostic or response-to-treatment) had the highest relative contribution to the explained-variation of the full model, which in other terms also had been experienced by the MAQC II consortium study [3]. The MAQC II study defined the difficulty of the classification problem as depending on the endpoint. Further, they found that data using a particular endpoint were easier to be classified than the same data when using other endpoints. It shows that the classification performance also depends on the difficulty of the classification problem. In clinical applications, the classification difficulty may also be related to the nature of the medical question: diagnostic, prognostic, or response-to-treatment. In a diagnostic question, the investigator tries to differentiate patients with or without the disease of interest, based on their gene expression. The prediction based on this type of problem should be less complicated than the other two, since the gene expression information is gathered at the time when the disease is already present or not. On the other hand, the response-to-treatment classification predicts an outcome that has to develop over time, based on the gene expression at the start of treatment. The future of a patient is determined by multiple factors, not only on the genomic factors when the information is gathered, but also all events between the information extraction and prediction time. Prognostic classification faces the same issue as response-to-treatment classification, and may be even more difficult. In our study, the classification difficulty is increasing from diagnostic to response-to-treatment. This finding has been experienced previously in Leukaemia [56], where the diagnostic classifier had higher classification performance than prognostic classification. Almost all their diagnostic classifiers had perfect results, while the best model for prognostic questions had only 78% accuracy. The effect of classification difficulty has also been observed by [57], which used the integrated Fisher score to rank the problem difficulty. The number of selected genes did not increase as the classification case became more difficult, which is confirmed by our results (Figure 3). Furthermore, they stated that the gene selection method had a low effect on the classification performance. In our result, the gene selection method was associated with the classification model performance. However, it is also worthy to note that we categorized the gene selection methods based on their interactions with the classifier. Dimensional reduction is often to be done by applying a particular gene selection technique before building a classification model. Some methods, however, have the ability to exclude redundant genes while building the classification model, e.g. PAM and LASSO. Unfortunately, the theoretical advantage of these methods was not used by [28], [43], [44], [48], [51], [55], making a gene selection step necessary before building the classification model. One reason for this might be because supervised learning and differentially expressed genes analysis were presented in the same paper.

The other important factor that should be considered in building a classification model is the cross-validation technique. It has the second largest of individual explained variability. The variability of classification performance could be due to the diversity of cross validation techniques that were used by investigators. It suggests that more attention shall be put to this factor when we build microarray classification models. Overoptimistic assessment of model performance is the most common flaw in class prediction studies, which causes an upward bias in estimates of prediction accuracy [1], [3]. Simple model evaluation is done by dividing the data into a training and a testing set, i.e. build a model in the training set and test the model into a dataset that is blinded for the training part. The test set should not be involved in any modelling step. An inappropriate approach is to first do the gene selection in the whole dataset and then make the split in training and testing set [1], [58], as had been done by [10], [18], [25], [32], [43]. In that case, the testing data was partly involved in a model building step through the selection of genes in the model, so that an overoptimistic classifier more likely will be produced. We found 18 studies (40%) that used cross validation for either model assessment or model selection, but not for both. Although our individual evaluation (Table S2 and Figure S2) shows that there is no difference in classification performance of both cross validation techniques, the multivariable final model showed that they do differ. Using cross validation for both model assessment and model selection is definitely to be advised in order to avoid producing an overoptimistic classifier. A framework for building classification models on gene expression data by [59] may be considered as a standard guide.

Another interesting finding in our study is the significant effect of microarray color system on model performance. The one-color microarray platform tends to yield higher accuracy than two-color systems (Figure S3). This result was contradictory with [60] who tested the dependence of the gene expression prediction in neuroblastoma patients on the microarray platform. They concluded that different microarray platforms should be able to yield similar results if the investigators follow the right procedure given by the vendor and they know the nature of those platforms.

Individual evaluation yielded no significant study factors that influenced the class prediction accuracy. A study factor may not be significant in a univariable model, but it may show its effect in a multivariable evaluation, due to the presence of other study factors in the meta-regression model, a mechanism well known as confounding. Hence, we also evaluated the study factors simultaneously by using backward selection. High associations were shown by the medical question and cross validation method (Table 5). The prognostic and response-to-treatment studies tended to use single cross validation method. Nested cross validation is difficult to use in small sample sized data. Hence, they might prefer to use single over nested cross validation. Meanwhile, most of the diagnostic classification studies used nested cross validation, where most of them had relatively large sample size. This explains the confounding in the individual analysis.

**Table 5:** The number of data points (average accuracy) clustered by “medical question” and “cross validation technique”.

		Medical question		
		Diagnostic	Prognostic	Response to treatment
**Cross validation technique**	Single	10 (0.93)	5 (0.90)	13 (0.83)
	Nested	27 (0.87)	2 (0.90)	4 (0.76)

Statistical power might be an issue in our study, due to relatively small number of selected studies used for analysis. However, it is also important to note that there are not much published gene expression classification studies outside the cancer field. We found forty eight studies that fulfilled all our requirements (detail search strategy is available in the Supplementary Material). Given the relatively small population of non-cancer published studies, our search yielded a considerable number of studies when it is compared to the literature review studies in the cancer field conducted by [1] (n = 90 studies) and [2] (n = 84 studies). Including cancer studies into our analysis might increase the statistical power and possibly lead to a difference in behaviour of the study factors in cancer and non-cancerous diseases. However, as aforementioned, gene expression studies in the field of cancer have been observed intensively by [1], [3], [4], particularly in the supervised learning case. Thus, in this study we chose to closely evaluate published classification studies in the microarray gene expression experiment outside the field of cancer. Although we have a relatively small number of studies, our finding in the multivariable evaluation have stable results, in which we found high agreement of the random effect logistic regression model in the jackknife resampling analysis and in the overall analysis (Supplementary Material, Table S3).

This study evaluated eight factors that represent the characteristics of the experiments as well as the gene expression data. The multivariable analysis shows that 42% of the between study variation was explained by these factors, while the other 58% might be explained by un-observed factors, which may include the preprocessing procedures (e.g. batch effect removal, normalization and filtering criteria), the microarray type, and the number of features after the preprocessing steps. We did not include factors such as normalization and batch effect removal due lacking information in the majority of the selected studies. This also rises a recommendation for each published gene expression study to report all steps both in the experiment and in the data analyses, as mentioned by the MIAME (Minimum Information About a Microarray Experiment) guideline [61].The transparency of study reporting helps to achieve reproducibility of the results and to serve as an input for further research, such as a meta-regression study. The un-reproducibility of result is even more severe when the datasets, in particular the raw dataset, are not publicly available [62]. Among our 48 selected studies, we found only eight studies that had stored both raw and processed datasets; and three studies that stored processed datasets only either in the ArrayExpress or the GEO online repository (last checked on February 4, 2014).

## Conclusions

The accuracy of classification models based on gene expression microarray data depends on study specific and problem specific factors. Investigators should pay more attention to these factors when building microarray classification models. The cross validation technique has an important impact in explaining the variability across the studies. Nested cross validation is suggested to be used in any microarray classification study.

## Supporting Information

Figure S1 **Plot of proportional sample size (the class imbalance level) and the classification model accuracy.** The class imbalance level was calculated by dividing the sample size in the majority class by the total sample size in the training data. The diagonal line represents a minimum accuracy that should be achieved by a classification model, based on assigning all subjects to the majority class.
(JPG)

Figure S2 **Boxplot of Cross Validation Technique against model accuracy.**
(JPG)

Figure S3 **Boxplot of microarray platform (color system) against model accuracy.**
(JPG)

Table S1 **Details of study factors in the selected studies.**
(XLSX)

Table S2 **The variability explained by each modeling factor.**
(DOCX)

Table S3 **Study factors that were included in the multivariable random effect logistic regression models via Jackknife resampling.**
(DOCX)

Checklist S1 **PRISMA checklist.**
(DOC)

Material S1 **Literature search strategy.**
(DOCX)

Material S2 **Disease classification.**
(DOCX)

Material S3 **R script.**
(DOCX)
