# Supplementary figures and images for: Evaluation of Gene Expression Classification Studies: Factors Associated with Classification Performance
Source: PLoS One. 2014 Apr 25;9(4):e96063. doi: 10.1371/journal.pone.0096063 (PMC4000205; doi:10.1371/journal.pone.0096063)

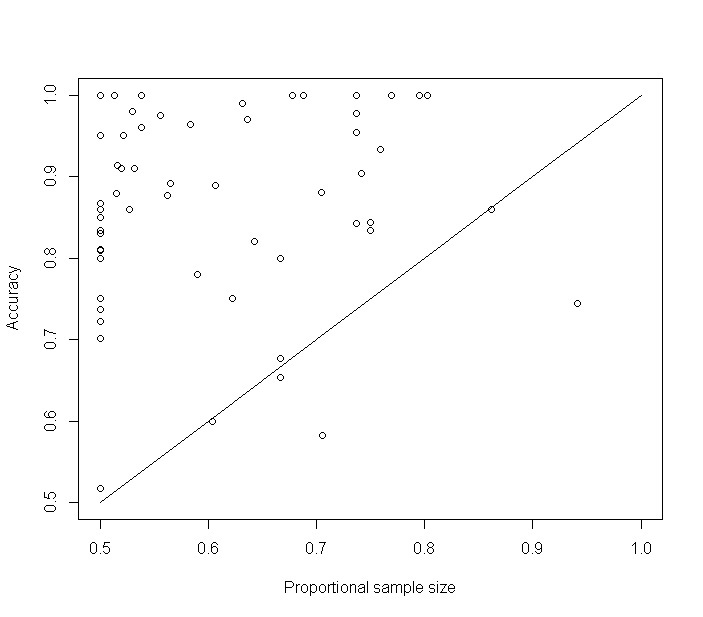

Supplement: Figure S1 — Plot of proportional sample size (the class imbalance level) and the classification model accuracy. The class imbalance level was calculated by dividing the sample size in the majority class by the total sample size in the training data. The diagonal line represents a minimum accuracy that should be achieved by a classification model, based on assigning all subjects to the majority class. (JPG) [file pone.0096063.s001.jpg]

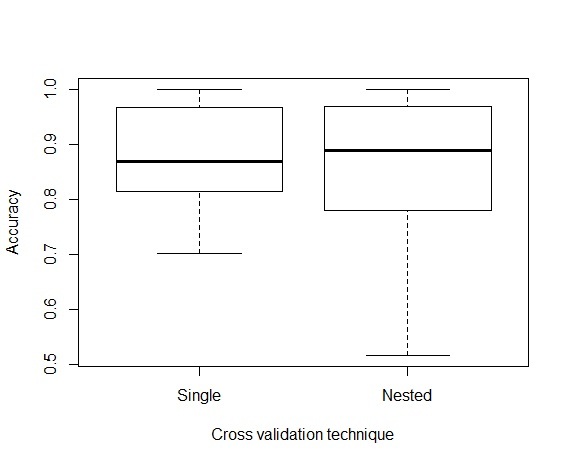

Supplement: Figure S2 — Boxplot of Cross Validation Technique against model accuracy. (JPG) [file pone.0096063.s002.jpg]

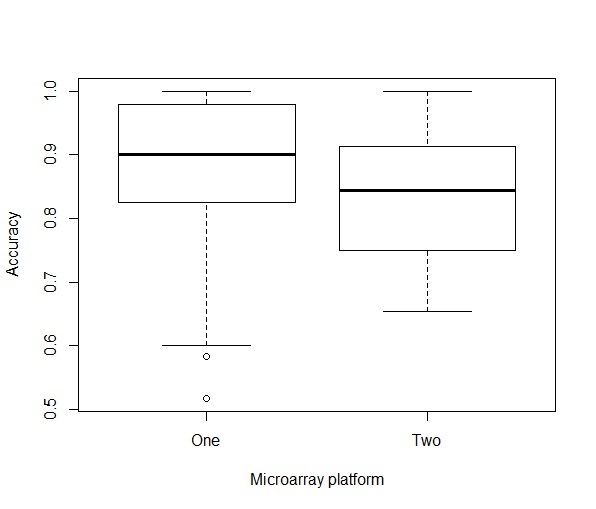

Supplement: Figure S3 — Boxplot of microarray platform (color system) against model accuracy. (JPG) [file pone.0096063.s003.jpg]
