# Supplementary material for: Evaluation of Gene Expression Classification Studies: Factors Associated with Classification Performance
Source: PLoS One. 2014 Apr 25;9(4):e96063. doi: 10.1371/journal.pone.0096063 (PMC4000205; doi:10.1371/journal.pone.0096063)
Supplement: Table S2 — The variability explained by each modeling factor. (DOCX) [file pone.0096063.s005.docx]

**Table S2. The variability explained by each modeling factor**

| **No** | **Variable** | **% Variability** |
| --- | --- | --- |
| 1 | Medical question | 24.89 |
| 2 | Cross validation techique | 9.23 |
| 3 | Disease group | 8.03 |
| 4 | Microarray color system | 2.48 |
| 5 | The number of genes | 1.85 |
| 6 | Gene selection technique | 1.32 |
| 7 | Sample size | 0.04 |
| 8 | Classification method | 0.02 |
