# Supplementary material for: Evaluation of Gene Expression Classification Studies: Factors Associated with Classification Performance
Source: PLoS One. 2014 Apr 25;9(4):e96063. doi: 10.1371/journal.pone.0096063 (PMC4000205; doi:10.1371/journal.pone.0096063)
Supplement: Table S3 — Study factors that were included in the multivariable random effect logistic regression models via Jackknife resampling. (DOCX) [file pone.0096063.s006.docx]

**Table S3**. Study factors that were included in the multivariable random effect logistic regression models via jackknife resampling

| **Study*** | **Study factors** | | | | | | | |
| --- | --- | --- | --- | --- | --- | --- | --- | --- |
|  | **N** | **CV** | **P** | **MedQues** | **Classif method** | **Color** | **Gene selection** | **Disease type** |
| Chen | - | v | - | v | - | v | v | - |
| Hakonarson | - | v | - | v | - | v | v | - |
| Koczan | - | v | - | v | - | v | v | - |
| Moore | - | v | - | v | - | v | v | - |
| Ockenhouse | - | v | - | v | - | v | v | - |
| Tan | - | v | - | v | - | v | v | - |
| Barth | - | v | - | v | - | v | v | - |
| Burczynski | - | v | - | v | - | v | v | - |
| Pachot | - | v | - | v | - | v | v | - |
| Allantaz | - | v | - | v | - | v | v | - |
| Kuo | - | v | - | v | - | v | v | - |
| Mutch | - | v | - | v | v | v | - | - |
| Tang | - | v | - | v | - | v | v | - |
| Wang | - | v | - | v | - | v | v | - |
| Ramilo | - | v | - | v | - | v | v | - |
| Ramilo2 | - | v | - | v | - | v | v | - |
| Aerssens | - | v | - | v | - | v | v | - |
| Cvijanovich | - | v | - | v | - | v | v | - |
| Koczan | - | v | - | v | - | v | v | - |
| Vahey | - | v | v | v | - | v | v | - |
| Xu | - | v | - | v | - | v | v | - |
| Arijs | - | v | - | v | - | v | v | - |
| Howrylak | - | v | - | v | - | v | v | - |
| Julia | - | v | - | v | - | v | v | - |
| Lin | - | v | - | v | - | v | v | - |
| Nascimento | - | v | - | v | - | v | v | - |
| Olsen | - | v | - | v | - | v | v | - |
| Popper | - | v | - | v | - | v | v | - |
| Tanino | - | v | - | v | - | v | v | - |
| Walter | - | v | - | v | - | v | v | - |
| Arijs | - | v | - | v | - | v | v | - |
| Fehlbaum-Beurdeley | - | v | - | v | - | v | v | - |
| Kabakchiev | - | v | - | v | - | v | v | - |
| Suárez-Fariñas | - | v | - | v | - | v | v | - |
| Takahashi | - | v | - | v | - | v | v | - |
| Woelk | - | v | - | v | - | v | v | - |
| Bansard | - | v | - | v | - | v | v | - |
| Scian | - | v | - | v | - | v | v | - |
| Glatt | - | v | - | v | - | v | v | - |
| Kong | - | v | - | v | - | v | v | - |
| Maschietto | - | v | - | v | - | v | v | - |
| Menke | - | v | - | v | - | v | v | - |
| Murakami | - | v | - | v | - | v | v | - |
| Rahimov | - | v | - | v | - | v | v | - |
| Rasimas | - | v | - | v | - | v | v | - |
| Swanson | - | v | - | v | - | v | v | - |
| Zhou | - | v | - | v | - | v | v | - |
| Balow Jr | - | v | - | v | - | v | v | - |
| Lunnon | - | v | - | v | - | v | v | - |

Table S3 shows the study factors that were included in the random effect logistic regression models if a particular study was excluded in each jackknife sample. The study factors that were included in the multivariable logistic regression model were indicated by “v”. Otherwise, it was presented by “-”.

* First author’s last name of the selected studies that were removed in each jackknife sampling

Abbreviation of the study factors (the detailed description of the study factors is available in the Method Section)

N : sample size in the training data

CV : cross validation technique

Medques : medical question

Classif method : classification method(s)

Color : microarray color system

Gene selection : gene selection technique

Disease type : disease classification of the selected studies
