# Supplementary material for: Evaluation of Gene Expression Classification Studies: Factors Associated with Classification Performance
Source: PLoS One. 2014 Apr 25;9(4):e96063. doi: 10.1371/journal.pone.0096063 (PMC4000205; doi:10.1371/journal.pone.0096063)
Supplement: Material S1 — Literature search strategy. (DOCX) [file pone.0096063.s008.docx]

**MATERIAL S1. LITERATURE SEARCH STRATEGY**

***Keywords***

- Main keywords

| **No** | **Key term** | **Sub term** |
| --- | --- | --- |
| 1 | Microarrays | micorarray* |
|  |  | gene expression |
| 2 | Classifications | classif* |
|  |  | predict* |
|  |  | supervise* |
| 3 | Evaluations | accura* |
|  |  | sensitifity |
|  |  | error rate |
|  |  | missclass* |
|  |  | validat* |
|  |  | success* |
| 4 | Gene related terms | expression* |
|  |  | mRNA* |
|  |  | transcript* |
| 5 | Outcomes | patient* |
|  |  | survival* |
|  |  | outcome* |
|  |  | probability* |
|  |  | prognos* |
|  |  | responder* |
|  |  | disease* |
|  |  | treatment* |
|  |  | chromosome* |
|  |  | response* |
|  |  | death* |
|  |  | blood* |

- Exclude papers from irrelevant journals

The published papers in the following journals were expected to contain only cancer-related and methodological papers.

*Cancer-related Journals*

1. Cancer research
2. Cancer
3. International journal of cancer. Journal international du cancer
4. Journal of the National Cancer Institute British journal of cancer
5. Gan to kagaku ryoho. Cancer & chemotherapy
6. Clinical cancer research : an official journal of the American Association for Cancer Research
7. Cancer letters "European journal of cancer (Oxford, England : 1990)
8. Cancer genetics and cytogenetics
9. Cancer chemotherapy and pharmacology
10. Breast cancer research and treatment
11. Cancer epidemiology, biomarkers & prevention : a publication of the American Association for Cancer Research, cosponsored by the American Society of Preventive OncologyZ Bulletin du cancer
12. Cancer treatment reports
13. Gan no rinsho. Japan journal of cancer clinics
14. Japanese journal of cancer research : Gann
15. Lung cancer (Amsterdam, Netherlands)
16. Cancer immunology, immunotherapy : CII
17. Leukemia : official journal of the Leukemia Society of America, Leukemia Research Fund, U.K
18. Leukemia & lymphoma
19. Leukemia research "Clinical lymphoma, myeloma & leukaemia
20. International journal of gynecological cancer : official journal of the International Gynecological Cancer Society
21. Cancer epidemiology, biomarkers & prevention : a publication of the American Association for Cancer Research, cosponsored by the American Society of Preventive Oncology
22. Cancer epidemiology
23. Journal of cancer epidemiology and prevention
24. Journal of cancer epidemiology
25. International journal of radiation oncology, biology, physics
26. Journal of clinical oncology : official journal of the American Society of Clinical Oncology
27. Gynecologic oncology
28. Annals of oncology : official journal of the European Society for Medical Oncology / ESMOZ International journal of oncology
29. Journal of surgical oncology
30. Oncology reports
31. Cancer epidemiology, biomarkers & prevention : a publication of the American Association for Cancer Research, cosponsored by the American Society of Preventive Oncology
32. Radiotherapy and oncology : journal of the European Society for Therapeutic Radiology and Oncology
33. Annals of surgical oncology
34. Zhonghua zhong liu za zhi [Chinese journal of oncology]Z Oncology
35. Journal of neuro-oncology
36. European journal of surgical oncology : the journal of the European Society of Surgical Oncology and the British Association of Surgical Oncology
37. Oncology (Williston Park, N.Y.)Z American journal of clinical oncology
38. Oncology nursing forum
39. Medical and pediatric oncology
40. Breast cancer research and treatment
41. Breast cancer research : BCRZ Breast cancer (Tokyo, Japan)
42. Clinical breast cancer
43. Breast cancer : basic and clinical research
44. Journal of breast cancer
45. Breast cancer : targets and therapy
46. Current breast cancer reports
47. BMC cancer
48. Molecular cancer therapeutics
49. Molecular cancer research : MCR
50. Molecular cancer
51. Tumori
52. Progress in experimental tumor research
53. Medical oncology and tumor pharmacotherapy
54. Brain tumor pathology
55. Journal of immunotherapy with emphasis on tumor immunology : official journal of the Society for Biological Therapy Rare tumors
56. Archivio italiano di patologia e clinica dei tumori
57. Noshuyo byori = Brain tumor pathology
58. Tumor research

*Methodological Journals*

1. Bioinformatics (Oxford, England)
2. BMC bioinformatics
3. Journal of bioinformatics and computational biology
4. Advances in bioinformatics
5. Conference proceedings : ... Annual International Conference of the IEEE Engineering in Medicine and Biology Society. IEEE Engineering in Medicine and Biology Society. Conference
6. IEEE transactions on bio-medical engineering
7. IEEE transactions on ultrasonics, ferroelectrics, and frequency control
8. IEEE transactions on image processing : a publication of the IEEE Signal Processing Society
9. IEEE transactions on neural networks / a publication of the IEEE Neural Networks Council
10. IEEE transactions on medical imaging
11. IEEE transactions on pattern analysis and machine intelligence
12. IEEE transactions on systems, man, and cybernetics. Part B, Cybernetics : a publication of the IEEE Systems, Man, and Cybernetics Society
13. IEEE engineering in medicine and biology magazine : the quarterly magazine of the Engineering in Medicine & Biology Society
14. IEEE transactions on visualization and computer graphics
15. IEEE transactions on information technology in biomedicine : a publication of the IEEE Engineering in Medicine and Biology Society
16. IEEE transactions on neural systems and rehabilitation engineering : a publication of the IEEE Engineering in Medicine and Biology Society
17. IEEE/ACM transactions on computational biology and bioinformatics / IEEE, ACMZ IEEE computer graphics and applications
18. IEEE transactions on nanobioscience IEEE transactions on rehabilitation engineering : a publication of the IEEE Engineering in Medicine and Biology Society
19. Proceedings / IEEE International Symposium on Biomedical Imaging: from nano to macro. IEEE International Symposium on Biomedical Imaging
20. IEEE transactions on nuclear science
21. Journal of computational biology : a journal of computational molecular cell biology
22. Journal of bioinformatics and computational biology
23. Artificial intelligence

- Exclude cancer studies

We added the following terms as exclusion criteria

1. oncology
2. cancer
3. tumour
4. tumor
5. neuroblastoma
6. lymphoma
7. carcinoma
8. leukaemia
9. leukaemia
10. ependymoma
11. adenocarcinoma
12. medulloblastoma
13. melanoma
14. rhabdomyosarcoma
15. glioma
16. retinoblastoma
17. mesothelioma
18. glioblastoma
19. pheochromocytoma
20. cholangiocarcinoma
21. AML
22. sarcoma
23. osteosarcoma
24. leiomyoma
25. leiomyosarcoma
26. myoma
27. myeloma
28. adenoma
29. lipoma
30. liposarcoma
31. leukemic
32. meningioma
33. astrocytoma
34. oligodendroglioma
35. chondrosarcoma

- Limit the publication date (2005-2013). Last search on September 20, 2013.
- Exclude non-English written publications.
- Exclude non-human microarray experiments.
- Exclude irrelevant papers that contain the following terms

1. tissue microarray
2. tissue array
3. protein array
4. comparative genomic hybridization
5. CGH
6. single nucleotide
7. SNP
8. cell line

- Exclude review papers.

***Steps and results***

We applied the main keywords by combining all the key terms by “*and*” and “*or*” for the variants of key terms.

*(microarray* OR "gene expression") (classif* OR predict* OR supervise*) (accura* OR sensitivity OR "error rate" OR missclass* OR validat* OR success*) (expression* OR mRNA* OR transcript*) (patient* OR survival* OR outcome* OR probability* OR prognos* OR responder* OR disease* OR treatment* OR chromosome* OR response* OR death* OR blood)*

Result: 10351 papers*

Next, the limitations and exclusion criteria were applied.

| **No** | **Exclusion or limitation criteria** | **Number of papers after applying the exclusion/limitation criteria** |
| --- | --- | --- |
| 1 | Irrelevant journals | 7452 |
| 2 | Cancer terms | 4175 |
| 3 | Publication date | 3402 |
| 4 | Limit to only in human experiments and published in English | 1931 |
| 5 | Special terms outside microarray/gene expression | 1812 |
| 6 | Review papers | 1546 |

The titles of the 1546 selected papers were screened manually, resulting in 419 papers. Then, the abstracts of the remaining papers were screened yielding 197 papers to be fully reviewed. In order to make the proposed statistical analyses possible, we determined the variables that should be mentioned in the selected papers (*see Methods Section in the main manuscript for the details of the extracted information*). Having fully reviewed the 197 paper, 57 studies were selected. However, the statistical analyses were applied to the 48 studies that mentioned, at least, “accuracy” as a performance measure.
