# Supplementary material for: Evaluation of Gene Expression Classification Studies: Factors Associated with Classification Performance
Source: PLoS One. 2014 Apr 25;9(4):e96063. doi: 10.1371/journal.pone.0096063 (PMC4000205; doi:10.1371/journal.pone.0096063)
Supplement: Material S2 — Disease classification. (DOCX) [file pone.0096063.s009.docx]

**MATERIAL S2. DISEASE CLASSIFICATION**

Thirty four different diseases were found among the 48 selected studies, namely

1. Acute cardiac allograft rejection
2. Acute infections caused by virus and bacteria
3. Acute lung injury
4. Alzheimer’s disease
5. Asthma
6. Autism spectrum disorder
7. Bowel syndrome
8. Crohn’s Disease
9. Cryopyrin-associated periodic syndromes
10. Dengue infection
11. Depression
12. Dilated cardiomyopathy
13. Dystonia
14. Facioscapulohumeral muscular dystrophy (neuromuscular disorder)
15. Hepatitis C
16. HIV/AIDS
17. Idiopathic arthritis
18. Ischemic stroke
19. Juvenile idiopathic arthritis
20. Kawasaki Disease
21. Lost kidney graft due to interstitial fibrosis and tubular atrophy
22. Obesity
23. Pneumonia
24. Psoriasis
25. Pterygia
26. Rheumatoid arthritis
27. Sarcoidosis
28. Schizophrenia
29. Sclerosis
30. Septic shock
31. Sepsis
32. Stroke
33. Thoracic Aortic Aneurysm
34. Ulcerative colitis

**Classification based on SNOMED**

The 34 aforementioned diseases were then classified by SNOMED (<http://eagl.unige.ch/SNOCat/>). We used the PubMed ID of each selected paper as an input. The SNOMED classification is a hierarchical classification, where the first output is a very specific disease. We tried to get higher parents and classified the diseases on the common parents. The result of the SNOMED classification is shown as follows.

1. Inflammatory disorder

- Rheumatoid arthritis
- Crohn’s Disease
- Ulcerative colitis
- Juvenile idiopathic arthritis
- Cryopyrin-associated periodic syndromes
- Sarcoidosis
- Pneumonia

1. Mental disorder

- Schizophrenia
- Autism spectrum disorder
- Depression

1. Infectious disease

- acute infections caused by virus and bacteria
- Dengue infection
- HIV

1. Systemic infection

- Sepsis

1. Disorder of immune function

- Sclerosis
- Kawasaki Disease

1. Obesity
2. Disorder of soft tissue

- Septic shock

1. Disorder of nervous system

- Alzheimer disease
- Dystonia

1. Disorder of skin

- Psoriasis

1. Disorder of cardiovascular system

- Stroke
- Ischemic stroke
- Dilated cardiomyopathy
- Thoracic Aortic Aneurysm

1. Disorder of liver

- Hepatitis C

1. Disorder of digestive system

- Bowel syndrome

1. Disorder of respiratory system

- Acute lung injury
- Asthma

1. Visual system disorder

- Pterygia

1. Muscular dystrophy

- Facioscapulohumeral muscular dystrophy

1. Not classified everywhere

- Lost kidney graft due to interstitial fibrosis and tubular atrophy,
- Acute cardiac allograft rejection

**Reclassified the diseases**

SNOMED did not only give the disease classification based on etiology, but also by the organ system or the location at which a disease occurs. We kept the etiological classes that resulted from the SNOMED classification, namely inflammatory disorder, disease of immune function, and infection. For diseases that were classified to the non-etiological classes, we re-grouped them into new classes as presented below. The diseases that were not classified by the initial classes are written in *italic* font.

1. Inflammatory disorder

- Rheumatoid arthritis
- Juvenile idiopathic arthritis
- Crohn’s Disease
- Ulcerative colitis
- Juvenile idiopathic arthritis
- Cryopyrin-associated periodic syndromes
- Sarcoidosis
- Pneumonia
- *Asthma*
- *Irritable Bowel syndrome*
- *Pterygia*
- *Lost kidney graft due to interstitial fibrosis and tubular atrophy*

1. Disease of immune function

- Sclerosis
- Kawasaki Disease
- *Psoriasis*
- *Acute cardiac allograft rejection*

1. Infection

- acute infections caused by virus and bacteria
- Dengue infection
- HIV/AIDS
- Septic shock
- Sepsis
- *Hepatitis C*
- *Dystonia*

1. Degenerative disease

- Stroke
- Ischemic stroke
- Dilated cardiomyopathy
- Thoracic Aortic Aneurysm
- *Alzheimer*
- *Facioscapulohumeral muscular dystrophy*

1. Mental disorder

- Schizophrenia
- Autism spectrum disorder
- Depression

1. Obesity *
2. Trauma

- Acute lung injury *

The diseases in Group 6 and 7 were then merged into the “other” group.
