# Supplementary material for: Evaluation of Gene Expression Classification Studies: Factors Associated with Classification Performance
Source: PLoS One. 2014 Apr 25;9(4):e96063. doi: 10.1371/journal.pone.0096063 (PMC4000205; doi:10.1371/journal.pone.0096063)
Supplement: Material S3 — R script. (DOCX) [file pone.0096063.s010.docx]

**MATERIAL S3. R SCRIPT**

In this sub section, we provided the R scripts to analyze the predetermined study factors (the details of the study factors are described in the Method Section and their values are depicted in the Table S1).

install.packages(‘lme4’)

library(‘lme4’)

# Estimate the number of samples that are correctly and incorrectly classified

# We treated the accuracy as a grouped binomial variable

# (see Method section for further details)

# acc = the accuracy of the classification model in the testing dataset;

# if it is not available, we used the accuracy in the training set.

# N = the sample size in the training set.

N.c = round(acc * N) #The number of correctly classified samples

N.m = N - N.c #The number of incorrectly classified samples

# Proportional sample size in training data (the class imbalance level)

n.prop = n.trainA /N #n.trainA: the sample size in class A

n.prop[which(n.prop<0.5)] = 1 - n.prop[which(n.prop<0.5)]

# ############################################################## #

# UNIVARIATE EVALUATIONS #

# ############################################################## #

# 0. NULL MODEL

model0 = glmer(cbind(N.c,N.m)~n.prop + (1|author),family=binomial)

summary(model0)

# 1. SAMPLE SIZE

model1 = glmer(cbind(N.c,N.m)~n.prop + N + (1|author),family=binomial)

summary(model1)

# 2. MICROARRAY PLATFORM (one- and two-color system)

model1 = glmer(cbind(N.c,N.m)~ n.prop+color+ (1|author),family=binomial)

summary(model1)

# 3. MEDICAL QUESTION (Diagnostic, Prognostic and Response-to-treatment)

model1 = glmer(cbind(N.c,N.m)~n.prop+ medques + (1|author),family=binomial)

summary(model1)

# 4. DISEASE TYPE

model1 = glmer(cbind(N.c,N.m)~n.prop+ disease_type + (1|author),family=binomial)

summary(model1)

# 5. CROSS VALIDATION TECHNIQUE

model1 = glmer(cbind(N.c,N.m)~n.prop+ cross_valid + (1|author),family=binomial)

summary(model1)

# 6. GENE SELECTION METHOD

model1 = glmer(cbind(N.c,N.m)~n.prop+ geneselect + (1|author),family=binomial)

summary(model1)

# 7. CLASSFICATION METHODS (interaction and no-interaction methods)

model1 = glmer(cbind(N.c,N.m)~n.prop+ classif + (1|author),family=binomial)

summary(model1)

# 8. THE NUMBER OF GENES IN FINAL MODEL (p)

model1 = glmer(cbind(N.c,N.m)~n.prop+ p + (1|author),family=binomial)

summary(model1)

# ############################################################## #

# MULTIVARIABLE EVALUATION #

# ############################################################## #

modelfull = glmer(cbind(N.c,N.m)~ n.prop + N + color + medques +

disease_type + cross_valid + geneselect + classif + p +

(1|author), family=binomial)

summary(modelfull)

drop1(modelfull,test="Chisq")

# The full model was then evaluated by the backward elimination approach

# The detail of the method is described in the Method section

# and the results are provided in the Table 4.
